# Mitochondrial Ultrastructural Alterations and Declined M_2_ Receptor Density Were Involved in Cardiac Dysfunction in Rats after Long Term Treatment with Autoantibodies against M_2_ Muscarinic Receptor

**DOI:** 10.1371/journal.pone.0129563

**Published:** 2015-06-18

**Authors:** Suli Zhang, Zhongmei He, Jin Wang, Li Wang, Ye Wu, Jie Wang, Tingting Lv, Huirong Liu

**Affiliations:** 1 Department of Physiology and Pathophysiology, School of Basic Medical Sciences, Capital Medical University, Beijing, 100069, P. R. China; 2 The Key Laboratory of Remodeling-related Cardiovascular Diseases, Capital Medical University, Ministry of Education, Beijing, 100069, P. R. China; 3 Department of Physiology, School of Basic Medical Sciences, Shanxi Medical University, Taiyuan, Shanxi, 030001, P. R. China; 4 Department of Pathology, School of Basic Medical Sciences, Shanxi Medical University, Taiyuan, Shanxi, 030001, P. R. China; 5 Department of Neurology, First Hospital of Shanxi Medical University, Taiyuan, Shanxi, 030001, P. R. China; 6 Beijing Key Laboratory of Metabolic Disorders Related Cardiovascular Diseases, Capital Medical University, Beijing, 100069, P. R. China; Harbin Medical University, CHINA

## Abstract

**Background:**

Previous studies showed that autoantibodies (M_2_-AA) against the second extracellular loop of M_2_ muscarinic receptor (M_2_AChR-el2) from dilated cardiomyopathy (DCM) serum could induce DCM-like morphological changes in mice hearts. However, the effects of M_2_-AA on the cardiac function during the process of DCM and the potential mechanisms are not fully known. The present study was designed to dynamically observe the cardiac function, mitochondrial changes, and M_2_ receptor binding characteristics in rats long-term stimulated with M_2_-AA *in vivo*.

**Methods:**

M_2_-AA-positive model was established by actively immunizing healthy male Wistar rats with synthetic M_2_AChR-el2 peptide for 18 months. Meanwhile, vehicle group rats were administrated with physiological saline. The change of mitochondrial membrane potential (ΔΨm) was detected by radionuclide imaging. The ultrastructure of mitochondria was observed under electron microscopy. The M_2_ receptor binding characteristics were determined by radioactive ligand binding assay.

**Results:**

After immunization for 12 months, compared with vehicle group, M_2_AChR-el2-immunized rats showed decreased myocardial contractility and cardiac diastolic function evidenced by declined maximal rate of rise of ventricular pressure and increased left ventricular end-diastolic pressure, respectively. Additionally, mitochondrial swelling and vacuolation were observed. At 18 months, M_2_AChR-el2-immunized rats manifested significant decreased cardiac systolic and diastolic function and pathological changes such as enlargement of right ventricular cavity and wall thinning; and the mitochondrial damage was aggravated. Furthermore, the M_2_ receptor maximum binding capacity (B_max_) of the M_2_AChR-el2-immunized rats significantly decreased, while the M_2_ receptor dissociation constant (K_d_) was increased.

**Conclusions:**

Our study suggested that long-term stimulation with M_2_-AA leaded to the ventricular dilatation and gradual deterioration of cardiac dysfunction. Mitochondrial damage and the down-regulation of M_2_ receptor density and affinity may be involved in the process.

## Introduction

Idiopathic dilated cardiomyopathy (DCM), a refractory and primary myocardial disease, is a major cause of death in patients with heart failure. However, the pathogenesis of DCM is not fully understood. Autoimmunity, viral infection, and genetic predisposition have long been considered as major causes for idiopathic DCM, among which autoantibodies have become one of the cornerstones in mediating the pathophysiology in these patients [1].

It has long been known that there are high titers of autoantibodies against the second extracellular loop of the M_2_ muscarinic receptor (M_2_-AA) in sera of patients with DCM [2]. Our previous study showed that both M_2_-AA positive rate and titer in the sera of 53 DCM patients were higher than those in 408 healthy subjects [3]. A previous study demonstrated that M_2_-AA could recognize the second extracellular loop of the M_2_ muscarinic receptor (M_2_AChR-el2) and stimulate the muscarinic receptor on rats’ heart membrane [4]. Recent studies also indicated that M_2_-AA was responsible for DCM-like morphological changes in mice that were immunized with M_2_ receptor for 8 weeks [5]. However, whether long term exposure to M_2_-AA can worsen cardiac function during progression of DCM remains largely unknown.

Metabolic disorder is one of the key mechanisms of cardiac dysfunction in DCM, which is mainly caused by mitochondrial injury. Existing evidence suggests that mitochondrial injury in a rat model of DCM is responsible for cardiac dysfunction [6]. A clinical study has also shown that mitochondrial dysfunction could aggravate the pathogenicity of DCM [7]. These reports suggest that mitochondrial injury plays a detrimental role in the pathogenesis of DCM. However, whether long-term stimulation by M_2_-AA could contribute to mitochondrial injury in myocardium of DCM is still undetermined.

M_2_ receptor is an essential molecule in cardiac function regulation (i.e. negative inotropy and chronotropy), which displays contrarian functions to those of β_1_-adrenoceptors. M_2_ receptor activation also plays important roles in regulation of mitochondrial function [8]. Studies have shown that like other G protein-coupled receptors, the expression and affinity of M_2_ receptor can be up- or down-regulated by a multitude of factors, such as agonist, antagonist [9], and stress [10]. Additionally, abnormal receptor function may cause weakened cardio-protective effects of M_2_ receptor. A previous study reported that M_2_-AA isolated from patients with DCM can directly activate M_2_ receptor and exhibit agonist-like effects [11]. However, whether changes of binding characteristics of M_2_ receptor are responsible for the M_2_-AA-induced cardiac dysfunction are still unknown.

In the present study, healthy rats were immunized with the synthetic peptide of M_2_AChR-el2 to clarify the pathological roles of M_2_-AA in DCM. We aimed to determine the progress of cardiac dysfunction, changes of mitochondrial membrane potential and ultrastructure, and M_2_ receptor binding characteristics in the M_2_-AA positive rat model.

## Materials and Methods

### Peptide Synthesis

The peptide-corresponding to the sequence of the second extracellular loop of the human muscarinic receptor-2 (residues 168–193, V-R-T-V-E-D-G-E-C-Y-I-Q-F-F-S-N-A-A-V-T-F-G-T-A-I-A, 95% purity) [4] was synthesized by Shanghai Biochemical Institute of Chinese Medical Science Academy using an automated peptide synthesizer. The peptides were purified by reverse phase HPLC on a C-18 column (Vydac, Hesporia, CA) and assessed on an automated amino acid analyzer (Beckman Instruments, CA).

### Immunization of animals

Ten-week old healthy male Wistar rats, lacking congenital M_2_-AA, were divided into two groups. The M_2_-AA group (n = 30) was immunized with a mixture of synthetic peptides corresponding to M_2_AChR-el2 and immunoadjuvants once a month for 18 months, and the vehicle group (n = 30) was immunized with a mixture of physiological saline and immunoadjuvants. The immunizing antigen was dissolved in 0.1 mol/L Na_2_CO_3_ solution (pH = 11.0). In the M_2_-AA group, rats were immunized based on body weight with 0.4 mg/kg of M_2_AChR-el2 peptide, which was emulsified in an equal volume of Complete Freund’s adjuvant (CFA) and injected subcutaneously. After a month, a booster injection of a mixture of the same dose of M_2_AChR-el2 antigen peptide with equal volume of Incomplete Freund’s adjuvant (IFA) was administered subcutaneously. CFA contains paraffin oil, mannide monooleate and killed mycobacterium tuberculosis, which can attract macrophages to the injection site and enhance immune response. On the other hand, IFA contains paraffin oil, mannide monooleate but lacks bacteria, therefore can minimize the side effects of adjuvant, and was thus used for the immunization boosts. The rats of the vehicle group received the mixture of physiological saline and adjuvant according to the procedure as described above. All rats were bled one day before each booster injection and sera collected. At the end of immunization, rats were executed to collect the hearts and sera samples. Carbon dioxide (CO_2_) inhalation was used for euthanasia of rats. The study conformed to AVMA Guidelines on Euthanasia and the Guide for the Care and Use of Laboratory Animals protocol published by the Ministry of the People's Republic of China and were approved by Shanxi Medical University Committee on Animal Care. During the study period, the rats were fed with rat chow and water ad libitum.

### Streptavidin-enzyme-linked immunosorbent assay (ELISA)

Blood samples were collected from the caudal veins pre-immunization every month. The sera was separated by centrifugation at 3000 g for 15 min, aliquoted and frozen at -20°C until analysis. The titers of M_2_-AA were dynamically measured by ELISA as reported previously [3], and the results were expressed as optical density (OD) values. The OD value was measured at 405 nm using an ELISA reader (Spectra Max Plus, Molecular Devices, Sunnyvale, California, USA). We also calculated the positive/negative (P/N) ratio [(the OD of sample—the OD of blank control) / (the OD of negative control—the OD of blank control)] of each sample. Negative control samples were prepared as follows: 100 sera samples from healthy rats with an OD value of less than 2.5 times the background OD were pooled and centrifuged at 1500 rpm for 10 min, and the supernatants were then divided into small aliquots and stored until use. Samples with a P/N value of ≥2.1 were considered as M_2_-AA-positive, and samples with a P/N value of ≤1.5 were considered as M_2_-AA-negative [12].

### Measurement of cardiac function *in vivo*

The rats were anesthetized with sodium pentobarbital (50 mg/kg, i.p.) and the left ventricle was catheterized with a Statham gauge (PE50) through the right common carotid artery. Then left ventricular function was measured and the data were recorded by the PowerLab data acquisition system (AD Instruments). The following primary and derived variables were continuously recorded on a beat-to-beat basis: left ventricular systolic pressure (LVSP), left ventricular end diastolic pressure (LVEDP), and maximal rate of rise and decline of ventricular pressure (±dp/dt_max_).

### Cardiac morphological and histopathological analysis

Histopathologic examination of the hearts was performed after routine fixation and paraffin embedding. The wall thickness, cavity dimension, and cavity area were measured at the mid-sections of each heart by a computerized image analysis system (MIAS-300, Sichuan, China).

Heart tissues were fixed with glutaraldehyde for transmission electron microscopy analysis. Briefly, small pieces of heart tissue were fixed with 2.5% glutaraldehyde in 0.1 mol/L phosphate buffer (pH 7.4) for 2 h at 4°C. After being washed with phosphate buffer, the specimens were post-fixed with 1% osmium tetroxide for 2 h at 4°C. Following dehydration with a graded series of acetone, the specimens were infiltrated with epoxies 618 ultra-thin sections (50 nm thicknesses) and cut using a LKB ultra-microtome IV after polymerization at 60°C, stained with uranyl acetate and lead citrate, and finally observed using a 100-CX transmission electron microscope (Japan).

### Myocardial radionuclide imaging

Myocardial radionuclide imaging was used to detect the mitochondrial membrane potential (ΔΨm) [13]. Myocardial uptake of ^99m^Tcmethoxyisobutylisonitrile (^99m^Tc-MIBI) was measured as heart and upper mediastinum (H/M) count ratio. The operation was performed as previously described [14]. Briefly, an 18.5-MBq dosage of ^99m^Tc-MIBI was slowly injected *via* rat tail vein and planar and single photon emission computed tomography views were obtained. Conventional gamma scintillation camera was utilized for image processing (Mobile Radioisotope Camera, Model BHP6602, Hamamatsu, Japan).

### Radio-ligand receptor binding assay (RBA)

Rats were sacrificed at 12 and 18 months after the initial immunization. Tissue preparation was performed using methods previously reported by Vandermolen *et al*. [15]. Briefly, left ventricles from each group were collected and rapidly frozen in liquid nitrogen, and then stored at -70°C until sectioning. Ventricular tissue was trimmed to a dimension of 5×5 mm and embedded in OCT media (Miles, USA). The tissue sections were consecutively cut 40 μM in thickness in a -18°C cryostat microtome (Ryocut 1800) with their serial order being noted. One of five consecutive sections, which were made into a set, was used to measure tissue protein *via* Bradford method to ensure that consecutive serial sections did not vary in size [16]. [^3^H]-quinuclidinyl benzylate ([^3^H]-QNB, 51 Ci/mmol, Amersham International, UK) was used as radioligand for M_2_-muscarinic receptor. Saturation-binding isotherms were obtained by incubating sections for 1h with varying concentrations of [^3^H]-QNB (0.125 nM—8 nM) at 26°C, and experiments performed in duplicate. The reaction was terminated by dipping slides into 0°C Tris buffer for 22 min. The slides were rapidly dried and each set of four consecutive sections was scraped from the slides with a blade into tubes respectively, and individual scintillation vials with 5 ml scintillation fluid (PPO 4 g, Popop 100 g, dissolved in 1000 ml xylol) was added to each vial and stabilized overnight and counted in a liquid scintillation counter (Beckman LS-3801) for muscarinic binding. Non-specific binding was determined in the presence of 10^–4^ mol/L atropine and amounted to less than 20% of total binding. Specific binding was obtained by subtracting non-specific binding from total binding. All binding data are given as specific binding. The saturation binding parameters B_max_ and K_d_ were determined using the Prism 2.01 Programs.

### Statistical analysis

All measured values are expressed as mean ± SEM. Data was analyzed by unpaired Student’s *t* test or ANOVA where appropriate. The analyses were carried out using SPSS 13.0 software. Statistical significance was set at *P* < 0.05.

## Results

### M_2_-AA-positive rat models were successfully established and resulted in dilated cardiomyopathy-like morphological features

**Fig 1:**
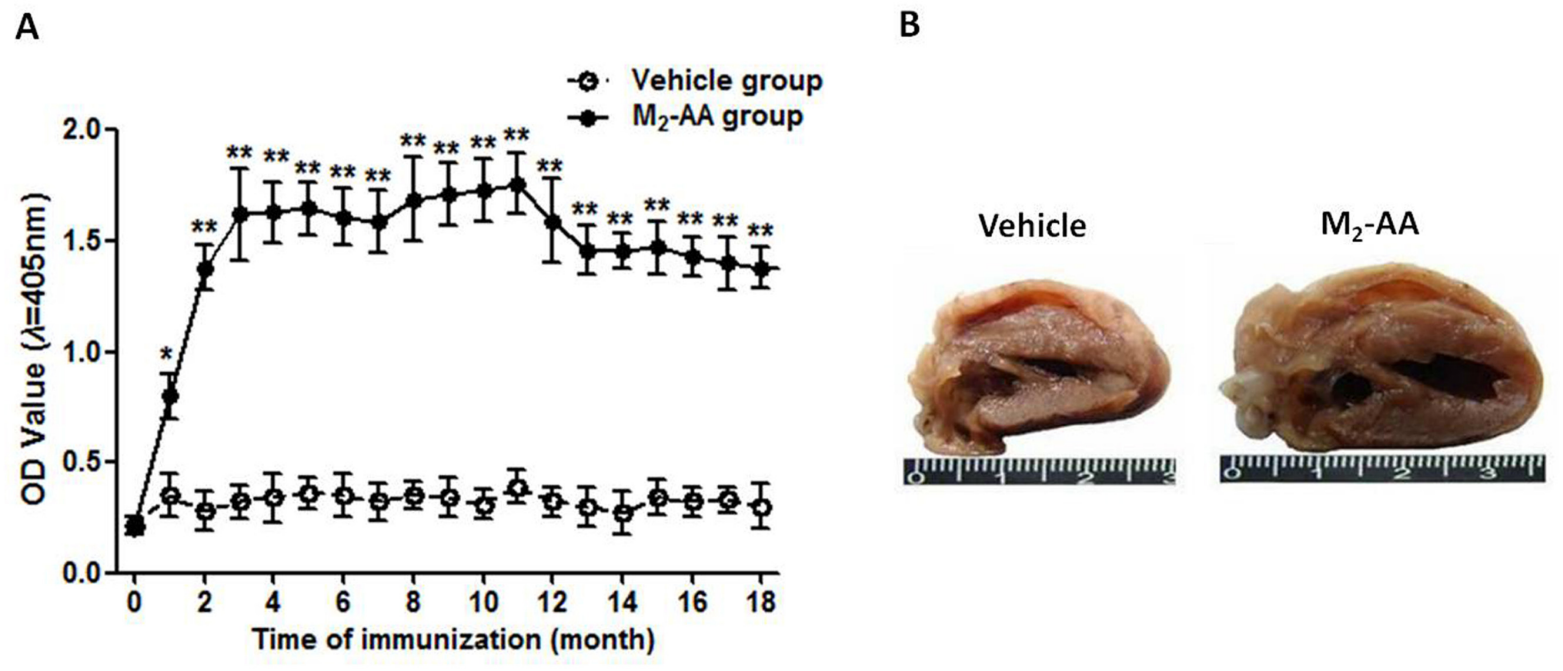
Establishment of M_2_-AA-positive rat models by actively immunizing with M_2_AChR-el2 antigen peptides for 18 months. (A) Time courses of M_2_-AA production during the process of active immunization in rats. Data are presented as mean ± SEM of optical density (OD) values measured by ELISA from three independent experiments at dilution of 1/640. The M_2_–AA group (n = 26) was immunized with a mixture of M_2_AChR-el2 antigen peptides and immunologic adjuvant, the vehicle group (n = 27) was immunized with a mixture of physiological saline and immunologic adjuvant. *P<0.05, **P<0.01 *vs*. vehicle group at the respective time-point. (B) Observation of the transverse sections of the hearts after 18 months of immunization with M_2_AChR-el2 antigen peptides. Hearts from the vehicle group showed normal chamber size and wall thickness, while hearts from the M_2_-AA group showed dilatation of the right ventricle with wall thinning. M_2_-AA, autoantibodies against M_2_ muscarinic receptor; M_2_AChR-el2, the second extracellular loop of M_2_ muscarinic receptor.

To determine the effect of long-term existence of M_2_-AA on cardiac structure and function *in vivo*, a M_2_-AA-positive rat model was established by active immunization with synthetic M_2_AChR-el2 antigen peptides for 18 months. Serum levels of M_2_-AA were detected by ELISA. In the immunization group, three rats (No. 3, 18 and 29) were euthanized after the 2^nd^ month for failing to generate M_2_-AA (P/N≤1.5). One rat (No.15) was euthanized due to inflammation at inoculation site after the 3^rd^ month. In the vehicle group, two rats (No.4, 16) were euthanized because of malocclusion induced-excessive weight loss and inflammation at inoculation site after the 2^nd^ and 3^rd^ month, respectively. One rat (No.30) died unexpectedly at the 4^th^ month. Therefore, there were 26 rats in the M_2_AChR-el2-immunized group and 27 rats in the vehicle group. During the immunization period, the M_2_-AA were positive (P/N≥2.1) in the M_2_AChR-el2-immunized group and maintained at a high level (Fig 1A), which peaked at the 11^th^ month (OD value, 1.76±0.14 *vs*. 0.39±0.07, *P*<0.01) and then declined gradually. The average titer of the antibodies during the 18 months was 1:1168.3±29.6. The Mg_2_-AA remained negative (P/N≤1.5) in the vehicle group throughout the observation period. The level and variance trends of M_2_-AA in the M_2_AChR-el2-immunized group were matched with the detected result of clinical patients, which indicates that this autoimmunity animal model was able to imitate clinical pathological progress.

The morphology of the hearts excised from the rats was observed post-immunization. As shown in Fig 1B, hearts from the M_2_-AA-positive immunization group showed moderate dilatation in the ventricles, including wall thinning. The dilatation was more apparent in the right ventricle (RV) than the left ventricle (LV), the RV/LV ratios of hearts from the M_2_-AA group increased significantly in cavity dimension and cavity area, while the wall thickness decreased significantly (Table 1) compared with the hearts from rats in vehicle group.

**Table 1:** RV/LV ratios from anatomic measurements of rat hearts at the end of the experiment.

Group	Wall thickness	Cavity dimension	Cavity area
Vehicle group	0.51±0.17	0.78±0.19	0.30±0.08
M_2_-AA group	0.42±0.07*	1.06±0.39**	1.51±0.24**

Data are presented as mean ± SEM (n = 5). RV: right ventricle; LV: left ventricle; M_2_-AA, autoantibodies against M_2_ muscarinic receptor.

*P<0.05.

**P<0.01 *vs*. control group.

### Long-term active immunization with M_2_AChR-el2 peptide induced cardiac dysfunction in rats

**Fig 2:**
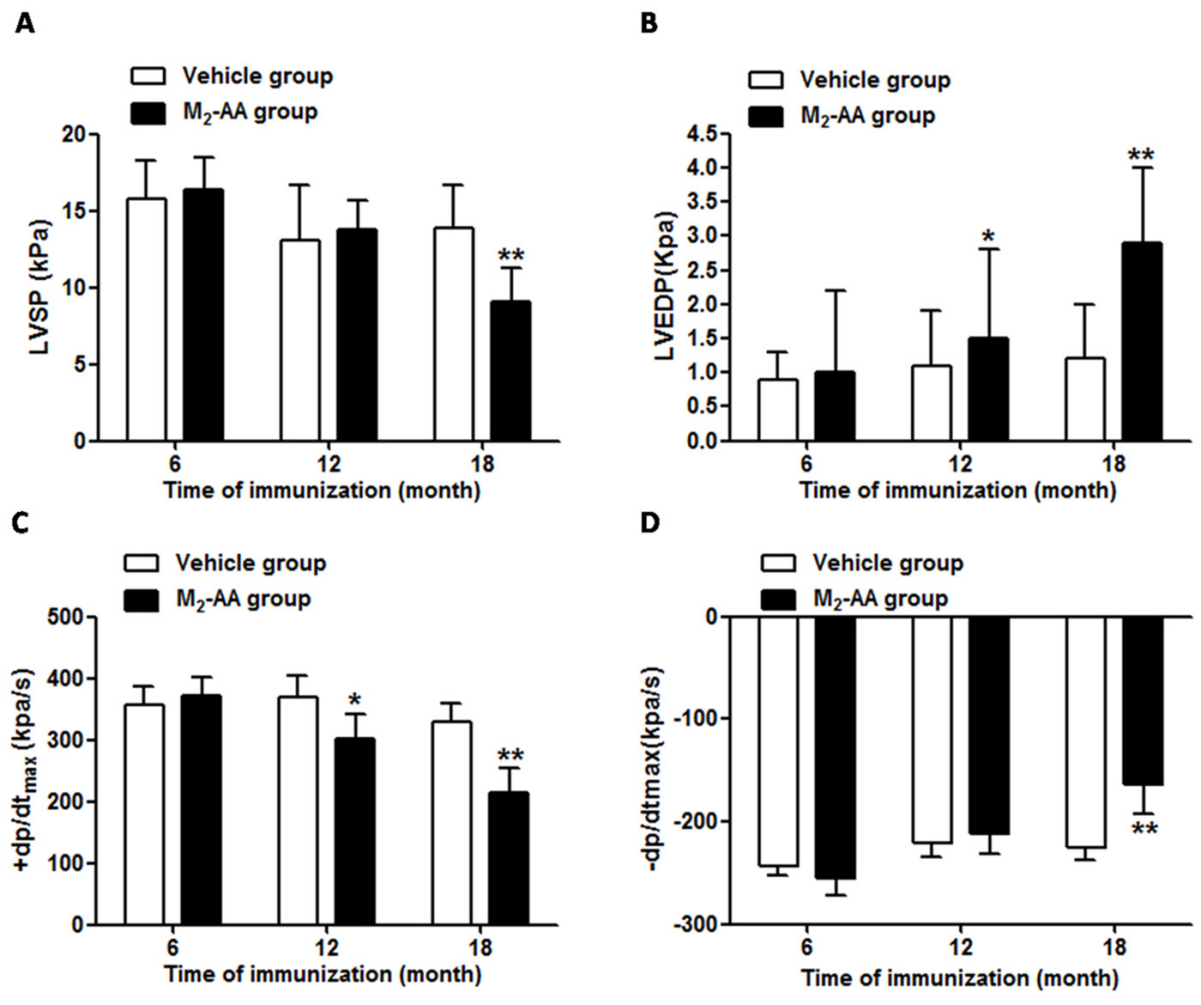
Dynamic monitoring of the left ventricular function changes of rats immunized with M_2_AChR-el2 for 18 months. Data are presented as mean ± SEM (n = 6). Panel A: left ventricular systolic pressure (LVSP, kPa); Panel B: left ventricular end diastolic pressure (LVEDP, kPa); Panel C: maximal rate of rise of left ventricular pressure (+dp/dt_max_, kPa/s); Panel D: maximal rate of decline of left ventricular pressure (-dp/dt_max_, kPa/s). *P<0.05, **P<0.01 *vs*. vehicle group at the corresponding time point.

To observe whether long-term active immunization with the M_2_AChR-el2 peptide influenced cardiac function in rats, hemodynamics parameters to evaluate left ventricular systolic and diastolic function were measured at 6, 12, and 18 months post-immunization. As shown in Fig 2, compared with the vehicle group, the left ventricular systolic pressure (LVSP) in the M_2_AChR-el2-immunized group had no significant change at the 12^th^ month, but markedly decreased at the 18^th^ month (Fig 2A). The left ventricular end-diastolic pressure (LVEDP) in the M_2_ group increased at the 12^th^ month (1.5±1.3 KPa *vs*. 1.1±0.8 KPa, *P*<0.05), and further increased at the 18^th^ month (2.9±1.1 KPa *vs*. 1.2±0.8 KPa, *P*<0.01) (Fig 2B). The maximal rate of rise 11 of left ventricular pressure (+dP/dt_max_) decreased at the 12^th^ month (302.4±39.0 KPa/s *vs*. 369.1±36.2 KPa/s, *P*<0.05) and decreased significantly at the 18^th^ month (213.0±40.3 KPa/s *vs*. 330.3±29.6 KPa/s, *P*<0.01) (Fig 2C). In addition, the maximal rate of decline of left ventricular pressure (-dP/dt_max_) increased significantly until the 18^th^ month (-163.6±28.4 KPa/s *vs*. -225.5±12.7 KPa/s, *P*<0.01) (Fig 2D). These results indicated that the cardiac function underwent no change in the first 6 months after M_2_AChR-el2 immunization; however, diastolic function and myocardial contractility of LV worsened at the 12^th^ month, and both the systolic and diastolic function of LV resulted in serious deterioration at the 18^th^ month post-immunization.

### Active immunization rats with M_2_AChR-el2 peptide caused a decrease in myocardial ΔΨm and structural changes of mitochondria

**Fig 3:**
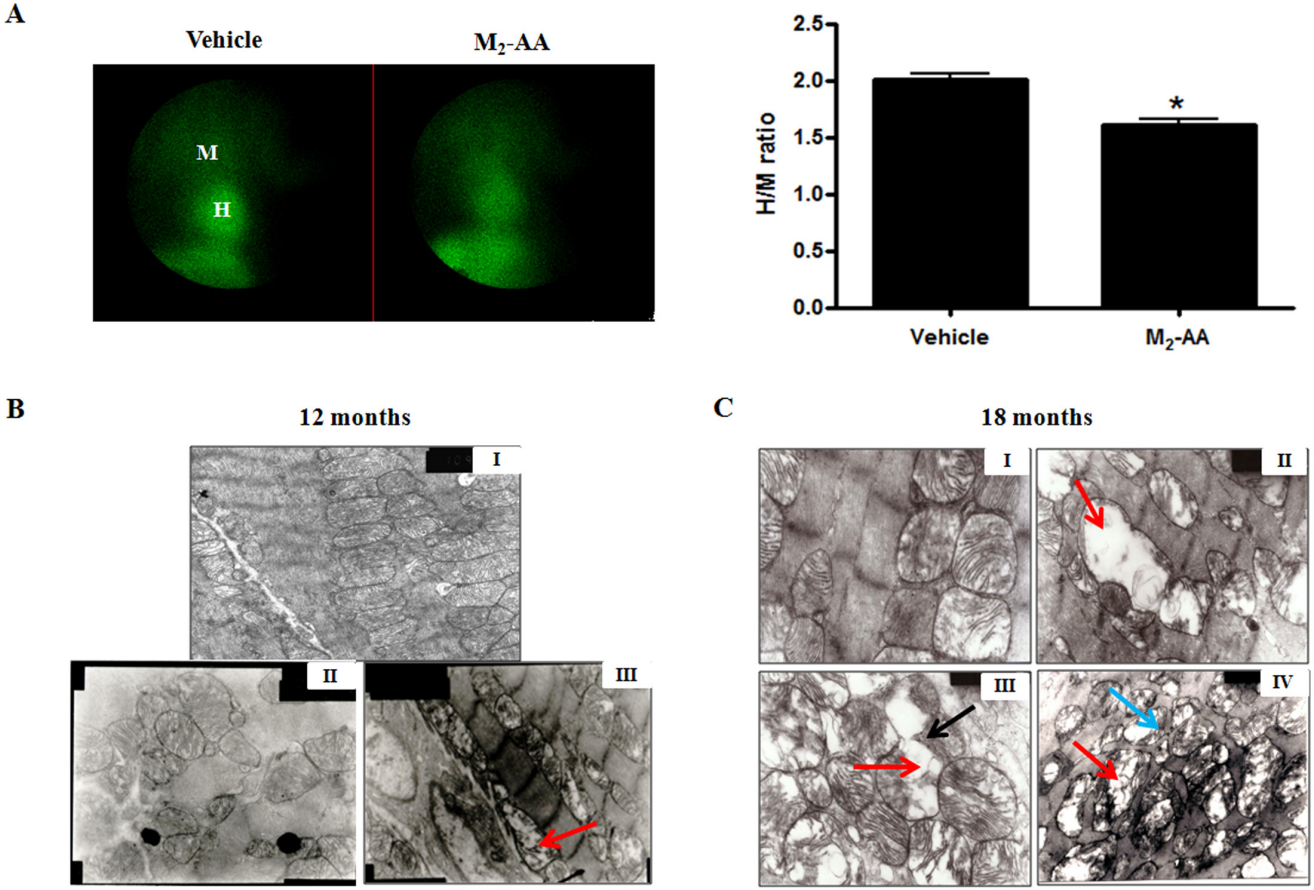
Changes of myocardial ΔΨm and mitochondrial ultrastructure during the active immunization process in rats. (A) Myocardial ^99m^Tc-MIBI uptake ratio of H/M in two groups of rats after 12 months of immunization. The H/M in M_2_-AA group rats decreased obviously compared to vehicle group rats. *P<0.05, *vs*. vehicle group. H/M, ^99m^Tc-MIBI uptake ratio of regions of interest positioned over the heart to mediastinum. Data are presented as mean ± SEM. n = 6/group. Alterations in the myocardial mitochondrial ultrastructure in two groups of rats after 12months (B) and 18 months (C) of immunization, respectively. Representative photomicrographs of the mid-papillary section of the heart from the vehicle group (panels B-I, C-II) and the M_2_ group (panels B-II~III, C-II~IV) were shown. Panels B-I~III, C-II, C-IV were photographed at ×10,000 magnification and panels C-I and C-III were photographed at ×20,000 magnification. The micrographs in M_2_-AA group displayed the irregular distribution of mitochondria, swollen mitochondria, vacuolar degeneration (as the red arrow directed), focal dissolved myofibrils (as the blue arrow directed), and deposition of dense granules in the sarcoplasm (as the black arrow directed).

Mitochondrial membrane potential (ΔΨm) is an important parameter of mitochondrial function [17]. Given that approximately 90% of myocardial ^99m^Tc-MIBI is localized within mitochondria and myocardial uptake of ^99m^Tc-MIBI is dependent on ΔΨm, the current study used radionuclide ^99m^Tc-MIBI myocardial perfusion imaging method to determine the alteration in ΔΨm as reported previously [14]. The regions of interest positioned over the heart (H) and upper mediastinum (M) represent the ^99m^Tc-MIBI uptake in the heart and mediastinum, respectively, and H/M uptake ratio can reflect the myocardial ΔΨm changes. As shown in Fig 3A, at the 12^th^ month post initial immunization, ^99m^Tc-MIBI uptake ratio of heart to mediastinum in M_2_-AA group rats decreased compared to vehicle group rats (1.63±0.05 *vs*. 2.07±0.09, *P<*0.05), which indicated that long-term exposure of M_2_-AA in rats could reduce nuclide uptake of heart and declined myocardial ΔΨm.

Along with the extension of immunity time, mitochondrial ultrastructural damage occurred in the M_2_AChR-el2-immunized group rats at 12^th^ month after initial immunization. From electron micrographs, myocardial specimens from the M_2_-AA group showed irregular distribution of mitochondria, varying degrees of mitochondrial swelling, cristae disappearance, and vacuolar degeneration (Fig 3B); moreover, the pathologic changes became more apparent at the 18^th^ month of immunization. Deposition of dense granules in sarcoplasm and focal-dissolved myofibrils were also observed at the end of the experiment (Fig 3C). On the other hand, the myocardial specimens from the vehicle group showed no obvious changes in mitochondrial ultrastructure.

### The influence of long-term stimulation of M_2_-AA on myocardial M_2_ receptor binding characteristics in rats

To study the influence of myocardial M_2_ receptor binding characteristics after long-term active immunization with M_2_AChR-el2 antigen peptide in rats, the largest combination capacity (B_max_) and dissociation constant (K_d_) of M_2_ muscarinic receptor were determined by RBA at the 12^th^ and the 18^th^ month post initial immunization. As shown in Table 2, in the M_2_-AA group, the B_max_ value reduced significantly at the 18^th^ month post M_2_AChR-el2-immunization compared to the vehicle group, and the K_d_ value was up-regulated at the 18^th^ month time-point compared to the vehicle group. These results indicated that the number of receptor sites were down-regulated by prolonged exposure to M_2_-AA; moreover, the remaining receptors were functionally declined.

**Table 2:** [^3^H]-QNB saturation binding parameters from rat heart homogenates at 12 and 18 months post-immunization.

Saturation bindings	B_max_ (fmol/mg protein)	K_d_ (nmol/L)
Vehicle group	73.8±10.1	3.90±0.42
M_2_-AA group (12th month)	82.8±9.8	5.37±0.38
M_2_-AA group (18th month)	49.2±5.7*	8.50±0.22*

Data are presented as mean ± SEM (n = 6). [^3^H]-QNB: [^3^H]-quinuclidinyl benzylate.

*P<0.05 *vs*. Vehicle group.

## Discussion

Dilated cardiomyopathy (DCM) is a cardiac disease characterized by ventricular cavity expansion and decline in contraction function. At present, etiology researches for DCM mainly focus on viral infection, cellular immunity, and autoimmunity; among which autoimmune mechanisms have received growing attention over the years [18]. The impact of autoimmune factors on heart disease has mainly focused on autoantibodies such as β_1_-AA and M_2_-AA [1]. In 1993, Fu *et al*. found that there was certain kind of autoantibody against M_2_AChR-el2 (M_2_-AA) in DCM patients, which suggested that autoimmune factors may participate in the development of DCM [2]. In our current study, a M_2_-AA positive rat model was established by method of active immunization with M_2_AChR-el2 and we found that: (1) long-term stimulation of M_2_-AA resulted in reduced myocardial contractility and diastolic function at the 12^th^ month, and both the systolic and diastolic dysfunction occurred at the 18^th^ month time-point; meanwhile, M_2_AChR-el2-immunized rats developed DCM-like pathological features, such as ventricular cavity expansion and wall thinning; (2) decreased mitochondrial membrane potential and mitochondrial ultrastructural damage were detected in M_2_-AA positive rats; and (3) the largest combination capacity (B_max_) and affinity (1/K_d_) of M_2_ receptors decreased in myocardial tissue of active immunization rat. In summary, this study indicates that the elevated M_2_-AA in the body could lead to myocardial mitochondrial damage, which may be one of potential causes for the decline in cardiac function and the compensatory expansion of ventricle which is similar to clinical features of DCM. Furthermore, down-regulation of M_2_ receptor density and affinity induced by M_2_-AA may also be involved in the process.

It has been shown that M_2_-AA, a subtype of IgG2a autoantibody [19], can bind to M_2_ receptors on cell surface and play a role similar to receptor agonist [2]. Wallukat G *et al*. observed a negative chronotropic effect of M_2_-AA on myocytes *via* M_2_ muscarinic receptor [20]. However, the pathophysiological roles of M_2_-AA in the development of DCM need further exploration. In the present model, long-term presence of M_2_-AA can lead to DCM-like morphological changes especially the right ventricular dilation, which is consistent with previous reports [5]. In addition, M_2_-AA can gradually deteriorate cardiac systolic and diastolic function. Cardiac catheterization is a classical method for detecting cardiac hemodynamics. The dp/dt refers to first-order differential of ventricular systolic pressure, which will generate the curve of pressure variation rate. The maximum of dp/dt (+dp/dt_max_) appeared in the first half of the isovolumic contraction period when preload and afterload are almost constant. Therefore, +dp/dt_max_ can be used as an important indicator to evaluate myocardial contractility under different functional statuses. Our study found that +dp/dt_max_ significantly declined at the 12^th^ month after initial immunization, which means that M_2_-AA depleted the myocardial contraction force. However, LVSP which reflects the peak ventricular systolic pressure was still within normal range due to compensatory mechanisms. Until the end of the immunization, LVSP in M_2_-AA group was significantly decreased, indicating ventricular systolic dysfunction (Fig 2A).

Ventricular diastole can be divided into two phases: active relaxation and passive stiffness. Ventricular active relaxation, mainly occurring in isovolumetric diastolic and rapid filling phases, needs to consume energy and can be represented by the change in diastolic pressure in the heart chamber per unit time (dp/dt). Ventricular passive stiffness mainly includes slow filling and atrial systolic phases during which no energy is needed. Either poor active relaxation or passive stiffness can induce elevated left ventricular end diastolic pressure (LVEDP). Additionally, LVEDP can be influenced by other factors, such as cardiac systolic force, heart rate, and intra-pericardial pressure, etc. Our study found that LVEDP increased at the 12^th^ month, and was further elevated at the 18^th^ month after initial immunization with M_2_AChR-el2 (Fig 2B), indicating the gradual deterioration of cardiac diastolic function. The reasons for the diastolic dysfunction are complex, and may include a longer filling time, weaken myocardial contractility, delayed cross-bridge detachment and Ca^2+^ dissociation from troponin C caused by energy shortage (evidenced by decreased—dp/d_max_ in Fig 2D), myocardial remodeling, and limited myocardial compliance.

It is well known that there are abundant myofibrils and mitochondria in myocardial cells. Mitochondrial damage can lead to abnormal energy metabolism and cardiomyocyte deaths, both of which can lead to weakened cardiac contractility and relaxation. In this study, to detect whether mitochondria was involved in cardiac disorder caused by M_2_-AA, myocardial mitochondrial membrane potential (ΔΨm) and ultrastructure changes were detected *via* radionuclide imaging and electron microscopy, respectively. Because of noninvasive examination, ^99m^Tc-MIBI perfusion imaging is widely used in clinical applications to diagnose and assess various cardiac diseases. As a free cationic complex, approximately 90% of ^99m^Tc-MIBI activity is associated with mitochondria in an energy-dependent manner *in vivo*. Previously, it has been shown that when mitochondrial membrane undergoes depolarization, uptake and retention of ^99m^Tc-MIBI by myocardium is inhibited. Hence the mitochondrial membrane condition can be reflected by the uptake rate of ^99m^Tc-MIBI [21]. Herein, we found that the ΔΨm evidenced by ^99m^Tc-MIBI uptake decreased in M_2_-AA-positive rats at 12 months post initial immunization, which is indicative of impaired mitochondrial function [17]. Moreover, electron micrographs showed that myocardial mitochondria displayed irregular distribution, swelling, and vacuolation. These pathological mitochondrial perturbations became more severe at the end of the process of immunization. Numerous studies have demonstrated that swollen mitochondrion [22], vacuolar degeneration [23], and altered myocardial ΔΨm [24] are widely used morphological and functional markers of abnormal mitochondria. These changes in mitochondria may lead to increased ROS formation, inhibition of mitochondrial respiratory chain, and eventually, cell death. While it is possible that attenuated cardiac systolic/diastolic function is due to abnormal energy metabolism from aberrant cellular activity as a result of mitochondrial damage, the exact mechanisms of M_2_-AA-induced mitochondrial damage remain to be studied. Given that cardiomyocytes entirely depend on aerobic oxidation for energy supply, identification of contributory factors that cause mitochondrial damage will undoubtedly be of great significance for myocardial salvage.

Previously, we showed that an autoantibody against the second extracellular loop of β_1_-adrenoceptor (β_1_-AA), contributed to the development of DCM [25]. Although both β_1_-AA and M_2_-AA have been shown to be involved in the development of DCM, they can lead to significantly different lesion characteristics. For example, (1) β_1_-AA-positive rat models established by active immunization exhibited elevated cardiac systolic and diastolic function at the 12^th^ month, which decreased significantly at the 18^th^ month after the initial immunization. On the other hand, cardiac dysfunction caused by M_2_-AA became more and more serious with the extension of immunization time. (2) Long-term existence of β_1_-AA caused mitochondrial condensation, while vacuolar degeneration represented the main feature of M_2_-AA-induced mitochondrial aberration. The data presented in the current study therefore suggests that M_2_-AA may trigger unique mechanism in cardiac injury. Moreover, there may be crosstalk between M_2_-AA and β_1_-AA, which could provide synergistic effects in causing declined cardiac function in DCM patients. It is also possible that the pathogenic role of M_2_-AA is prior to that of β_1_-AA. Notably, Stavrakis *et al*. recently demonstrated that M_2_-AA could neutralize the contractive effect of β_1_-AA on isolated Purkinje fibers [26], which further complicates the relationship between M_2_-AA and β_1_-AA. Therefore, the detailed interplay of β_1_-AA and M_2_-AA in DCM is worthy of further investigation.

M muscarinic receptors are cholinergic receptors that are widely present in humans. They are divided into three types, namely M_1_, M_2_, M_3_ muscarinic receptor depending on their different expression sites and function. Among them, M_2_ muscarinic receptor is mainly distributed in the heart and negatively regulates heart rate and contractile forces of cardiomyocytes, which balances with the roles of the β-adrenoceptor. In this study, specific ligands were bound directly to frozen heart tissue embedded on slides in order to measure maximal binding sites and affinity of specific receptors on cardiomyocytes in a manner that closely mimics *in vivo* condition. At the end of immunization, we found that M_2_ receptor maximum binding capacity (B_max_) decreased in M_2_-AA active immunization model of DCM, suggesting a decrease in the expression of M_2_ receptors; meanwhile, the dissociation constant (K_d_) standing for M_2_ receptor-ligand binding affinity was increased, implying a declined function of the available receptors. Receptor endocytosis is a significant mechanism of receptor degradation which can lead to decreased density of membrane receptor [27]. Delaney K.A, *et al*. reported that upon agonist stimulation, M_2_ receptor was internalized *via* clathrin-independent pathway and resulted in decreased receptor density [28]. In our study, long term stimulation of M_2_ receptor by M_2_-AA may be the cause of decreased M_2_ receptor density. The down-regulated affinity may be due to the changes of receptor conformation [29]. However, whether receptor endocytosis and conformational changes are critical mechanisms underlying reduced receptor binding ability need to be examined further. Additionally, associated signal pathways remain to be explored. However, studies have found that the inactivation of M muscarinic receptor plays an important role in cell apoptosis induced by mitochondrial injury or dysfunction [9]. This implies that decreased M_2_ receptor binding ability may be involved in mitochondrial damage, which could further contribute to cardiomyocyte damage. Specifically, future studies need to examine whether M_2_-AA can directly induce mitochondrial injury through interfering with M_2_ receptor.

Unexpectedly, a disturbance in the ratio of CD4^+^ and CD8^+^ T cells was also observed during the process of M_2_-AA-induced DCM in rats (S1 Fig). CD4^+^ and CD8^+^ are two subtypes of T lymphocytes which usually maintain a stable ratio under normal conditions, and often, increases or decreases in this ratio is indicative of autoimmune disorders. In this study, the CD4^+^/CD8^+^ ratio increased during the three months post-immunization, and decreased gradually after that. The increased CD4^+^/CD8^+^ ratio implied autoimmune hyperactivity during which more autoantibodies could be produced. This speculation was verified by the direct effect of M_2_-AA on the proliferation of T lymphocytes *in vitro* (S2 Fig). However, the decreased CD4^+^/CD8^+^ ratio indicated the immune suppression. Taken together, long-term stimulation of M_2_-AA can result in more severe immune imbalance; a likely vicious circle which should be further explored in the future studies.

### Study limitations

In this study, we have observed that long-term exposure to M_2_-AA results in DCM-like cardiac structural and functional changes, mitochondrial damage, and decreased M_2_-AA receptor density and affinity; however, more *in vivo* and *in vitro* experiments are needed in our further studies to understand the causal relationship between them and the potential mechanisms.

## Supporting Information

S1 Fig Ratios of CD4^+^/CD8^+^ T lymphocyte subsets pre- and post-immunization with M_2_AChR-el2 antigen peptides. (PDF)

S2 Fig The effect of M_2_-AA on proliferation of cultured rat T lymphocyte *in vitro*. (PDF)
