# Supplementary material for: Mitochondrial Ultrastructural Alterations and Declined M2 Receptor Density Were Involved in Cardiac Dysfunction in Rats after Long Term Treatment with Autoantibodies against M2 Muscarinic Receptor
Source: PLoS One. 2015 Jun 18;10(6):e0129563. doi: 10.1371/journal.pone.0129563 (PMC4472961; doi:10.1371/journal.pone.0129563)
Supplement: S1 Fig — (PDF) [file pone.0129563.s001.pdf]

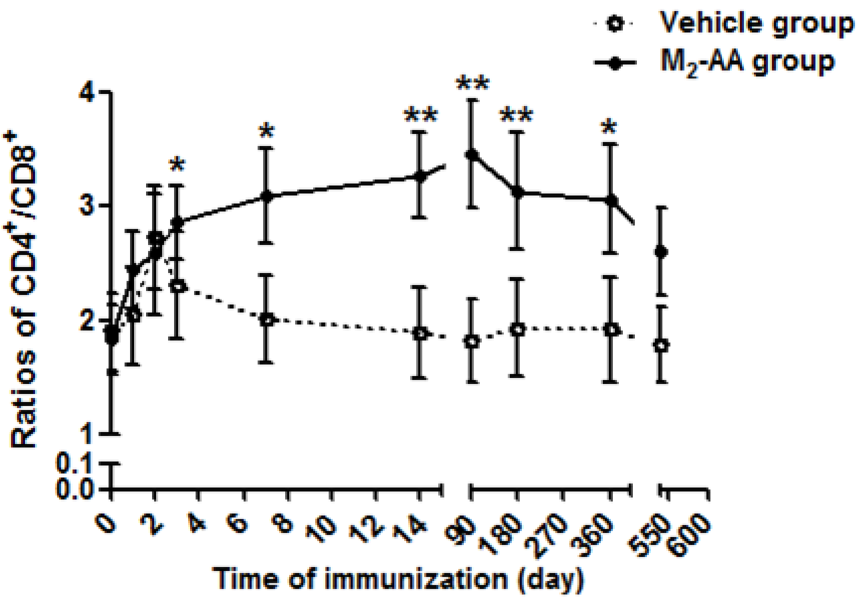

S1 Figure. Ratios of $CD4^+/CD8^+$ T lymphocyte subsets pre- and post-immunization with M_2AChR-el2 antigen peptides. Data are presented as mean ± SEM. $CD4^+/CD8^+$ represent ratio of helper T cells to cytotoxic T cells. n=4/group. $^*P<0.05$, $^{**}P<0.01$ *vs.* vehicle group at the respective period. M_2-AA, autoantibodies against M_2 muscarinic receptor.
