# Supplementary material for: Mitochondrial Ultrastructural Alterations and Declined M2 Receptor Density Were Involved in Cardiac Dysfunction in Rats after Long Term Treatment with Autoantibodies against M2 Muscarinic Receptor
Source: PLoS One. 2015 Jun 18;10(6):e0129563. doi: 10.1371/journal.pone.0129563 (PMC4472961; doi:10.1371/journal.pone.0129563)
Supplement: S2 Fig — (PDF) [file pone.0129563.s002.pdf]

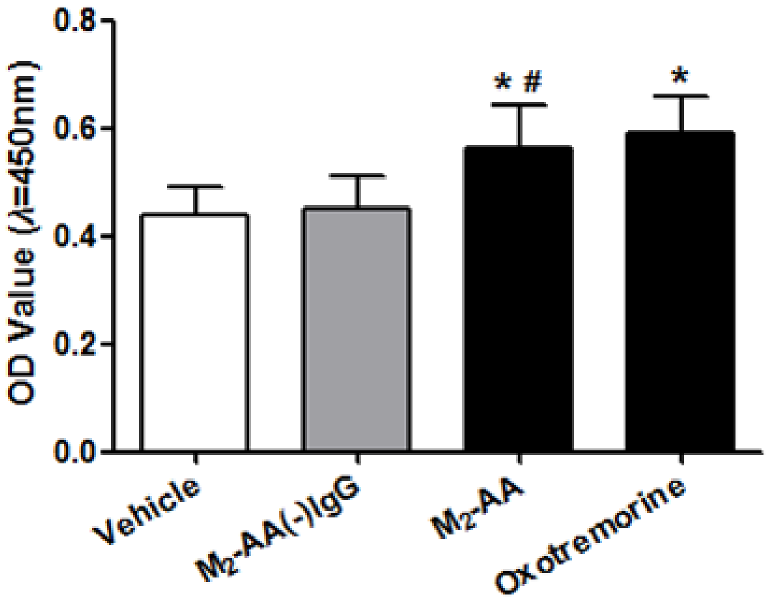

S2 Figure. The effect of M_2-AA on proliferation of cultured rat T lymphocyte in vitro. The viability of T lymphocyte was detected by CCK8 assay. Both M_2-AA (0.1 μmol/L) and M_2 receptor agonist oxotremorine (0.1 μmol/L) can promote the proliferation of T lymphocyte after treatment for 24 h. n=6/group. $^{*}P<0.05$ *vs.* Vehicle. $^{\#}P<0.05$ *vs.* M_2-AA (-) IgG. M_2-AA, autoantibodies against M_2 muscarinic receptor; M_2-AA (-) IgG, M_2-AA-negative IgG purified from healthy rat sera.
